# Retraction Note: Circular RNA circ_001621 promotes osteosarcoma cells proliferation and migration by sponging miR-578 and regulating VEGF expression

**DOI:** 10.1038/s41419-025-08326-0

**Published:** 2025-12-12

**Authors:** Xianglu Ji, Liping Shan, Peng Shen, Ming He

**Affiliations:** 1 Department of Orthopedic Surgery, Shengjing Hospital of China Medical University, Shenyang, Liaoning PR China; 2 Department of Urology Surgery, Shengjing Hospital of China Medical University, Shenyang, Liaoning PR China

Retraction Note to: *Cell Death & Disease* 10.1038/s41419-019-2204-y, published online 06 January 2020

The Editors in Chief retracted this article because of significant concerns regarding a number of Figures presented in this work, which question the integrity of the data. After publication, partial overlap and high similarities between figures were detected. Specifically, between Fig 3B and Fig 5B, Fig 4C of this paper and Fig 4F in an article published earlier [1], Fig 3D of this paper and Fig 2E in another article published earlier [[2], now retracted].

In addition, the authors did not respond to request to provide evidence of ethic approval for either animal or human part of the study presented here, or if consent has been obtained for guardians or parents of the underage participants.

The Editors-in-Chief therefore no longer have confidence in the integrity of the data in this article.

The Authors did not respond to correspondence from the Publisher about this retraction.
